# Rapid Airway Narrowing Associated with Hodgkin’s Lymphoma, a Case Report

**DOI:** 10.21980/J86D3Q

**Published:** 2020-04-15

**Authors:** Luke Hoffmann, Toby Myatt

**Affiliations:** * University of California, Irvine, Department of Emergency Medicine, Orange, CA

## Abstract

Hodgkin’s lymphoma is a neoplastic proliferation of Reed Sternberg cells and inflammatory cells within the lymphatic system. Common symptoms associated with Hodgkin’s Lymphoma include pronounced lymphadenopathy, fever, polynuclear leukocytosis, and anemia.^1^,^2^ This case study presents a unique case where an adult patient with nodular sclerosing Hodgkin’s lymphoma (NSHL) presents with rapidly progressing dyspnea due to tracheal airway narrowing. CT results showed pathological lymph nodes in bilateral cervical stations, subclavian images and within the mediastinum, concerning for lymphoproliferative disease. Otolaryngology was consulted for a possible tracheostomy; however, an awake fiberoptic intubation was suggested and subsequently performed instead. This case demonstrates the importance of immediate intervention to prevent airway loss in an atypical presentation of NSHL.

**Topics:**

Nodular sclerosing Hodgkin’s lymphoma, airway loss, intubation.

[Fig f1-jetem-5-2-v11]

**Figure f1-jetem-5-2-v11:**
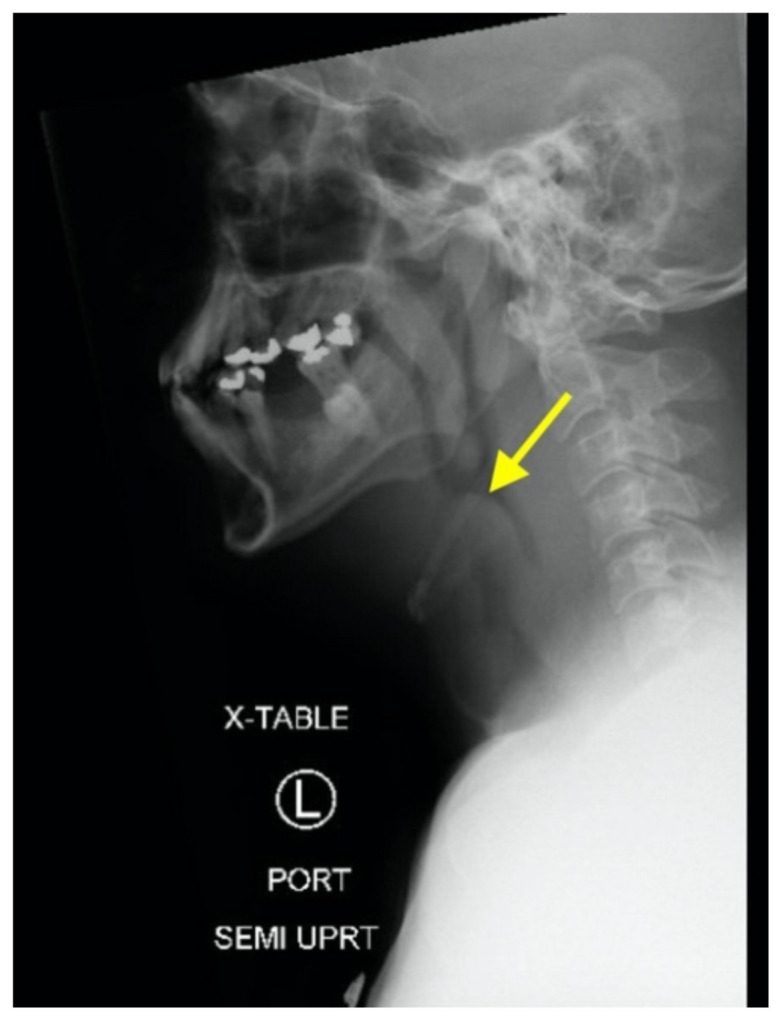

[Fig f2-jetem-5-2-v11]

**Figure f2-jetem-5-2-v11:**
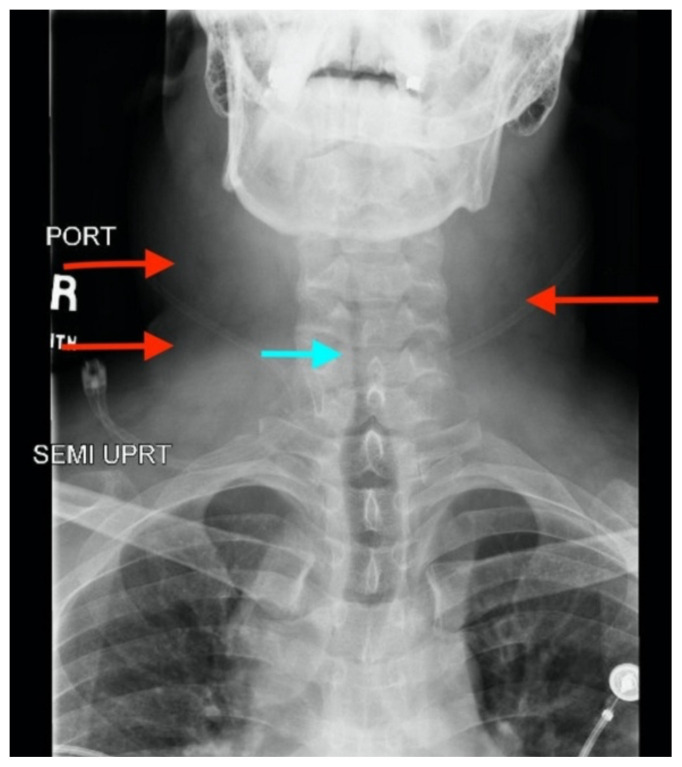

[Fig f3-jetem-5-2-v11]

**Figure f3-jetem-5-2-v11:**
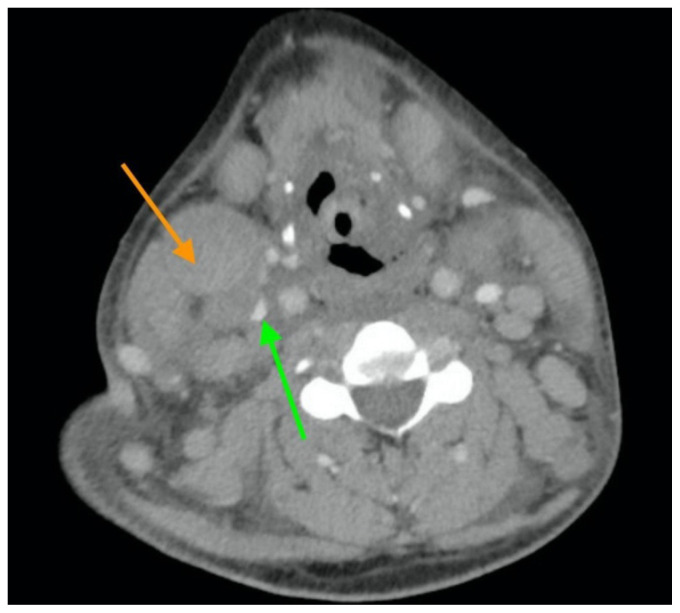

[Fig f4-jetem-5-2-v11]

**Figure f4-jetem-5-2-v11:**
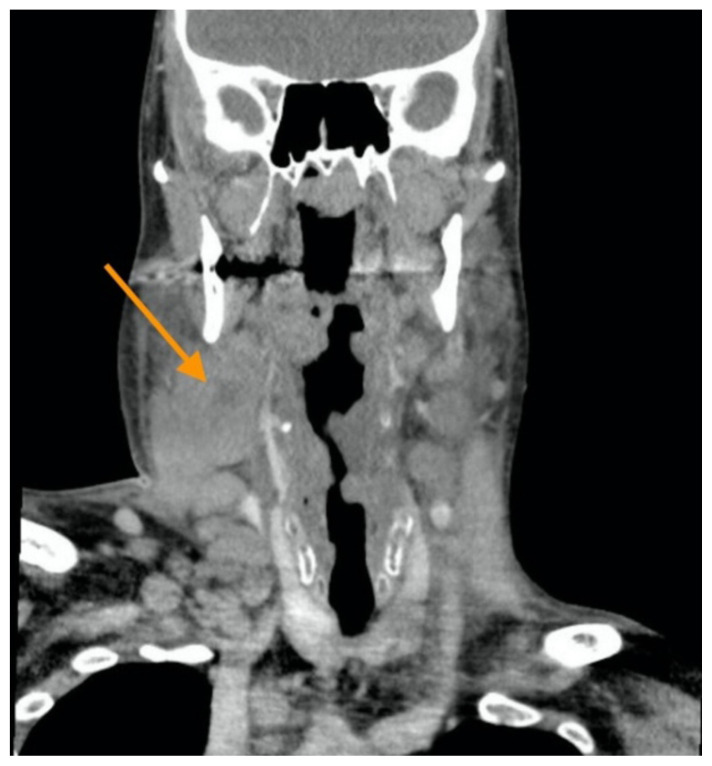

## Introduction

Nodular sclerosing Hodgkin’s lymphoma is associated with neck swelling due to severe lymphadenopathy in the cervical, supraclavicular, and mediastinal lymph nodes.^3^,^4^ Although rare in adults, the proliferating lymph nodes in Hodgkin’s lymphoma can lead to rapid airway narrowing. Immediate recognition of the airway compromise and intervention with intubation is essential to protect the patient’s airway without having to perform a tracheostomy or cricothyrotomy.

## Presenting concerns and clinical findings

A 36-year-old male presented to the emergency department (ED) with worsening dysphagia and dyspnea. The patient has a medical history significant of atopic dermatitis and NSHL. The patient was recently diagnosed with NSHL one week prior to reporting to the ED. The vital signs showed a blood pressure of 131/69, pulse of 120, temperature of 98.8°F, and a respiratory rate of 18. The initial neck examination showed neck swelling with moderate to severe lymphadenopathy, predominating on the right side of the neck. The pulmonary exam was normal with no audible stridor and the lungs were clear to auscultation bilaterally.

## Significant findings

Neck X-ray showed nonspecific significant prevertebral soft tissue swelling at the level of the cervical spine, with associated apparent thickening of the epiglottis (yellow arrow), diffuse soft tissue swelling of the neck (red arrows) and tracheal airway narrowing (light blue arrow). The computed tomography imaging of the neck was significant for multiple conglomerating pathological lymph nodes with a significant mass effect (orange arrows) compressing the right internal jugular vein (green arrow).

## Patient course

Otolaryngology (ENT) was consulted and evaluated the patient and noted significant supraglottic edema secondary to venous congestion from bulky lymphadenopathy, resulting in airway compromise. The patient was emergently transported to the operating room for an awake fiberoptic intubation where the procedure was successfully performed without a tracheostomy. Following the procedure, the patient was admitted to the intensive care unit (ICU) and was able to be extubated after ten days following courses of steroids, antibiotics, and diuresis. Patient was discharged with the plan to follow-up in clinic to obtain tissue slides to confirm diagnosis, complete staging, and begin treatment for NSHL.

## Discussion

Malignant lymphomas of the neck are divided into two groups, non-Hodgkin’s lymphoma (NHL) and Hodgkin’s lymphoma (HL). These lymphomas are distinguishable with tissue biopsies because HL will present with Reed-Sternberg cells while NHL does not.^5^ Within the subtypes of HL the Nodular Sclerotic subtype is the most common because it occurs approximately 70 percent of the time and has a good prognosis with chemotherapy with or without radiation.^6^

In our patient’s case, the neck swelling from the severe lymphadenopathy is a common manifestation of NSHL but the resulting airway narrowing is an abnormal finding. In adults, the lymphomatous nodes are soft compared to the strong and durable cartilaginous skeleton of the trachea. This anatomical feature of the trachea prevents compression by the adjacent enlarged lymph nodes. Our patient’s dyspnea due to airway narrowing is a symptom predominately found in children with HL; children diagnosed with HL frequently present with respiratory complications because their tracheal and proximal bronchial cartilage are pliable and easily compressed by the nearby lymph nodes.^7^,^8^ Our patient’s airway compromise may have also been partially a result of the lymph nodes compressing nearby vasculature, resulting in severe venous congestion causing diffuse facial swelling and placing excess pressure on the airway.

A CT scan was ordered for the patient to determine why he was having worsening dysphagia and dyspnea. Although airway narrowing in a patient with NSHL is an abnormal finding, any patient with throat tightness, stridor, dysphagia, or respiratory distress should be sent for a CT to evaluate the source of the symptoms. In some cases, NHL can even present with airway obstruction, especially when patients have primary tracheobronchial lymphomas, and would also require a CT to evaluate the cause of the respiratory distress.^9^

In the emergency department, nasotracheal intubation is considered for angioedema of the tongue, cervical spine injuries, mechanical obstructions that prevent the mouth from opening, as well as any structural abnormality that would make a traditional intubation difficult.^10^ Nasotracheal intubation can be performed blind or with fiberoptic assistance but oftentimes blind nasotracheal intubation is only performed if fiberoptic nasotracheal intubation is not available.^11^ In our patient’s case, awake fiberoptic nasotracheal intubation was chosen because we did not want to sedate him or take away his ability to breathe on his own in case the intubation failed.

The patient’s airway narrowing and rapidly progressing dyspnea are atypical findings associated with NSHL. The medical team caring for the patient was able to recognize this abnormal presentation and take the patient to the operating room for an emergency awake nasal intubation. The decision to immediately intubate the patient prevented the need for an emergency tracheostomy or cricothyrotomy and underscores the importance of early airway intervention in patients with rapidly progressing airway compromise.

## Supplementary Information

















## References

[1] Hodgkin’s lymphoma (Hodgkin’s disease) - symptoms and causes Mayo Clinic https://www.mayoclinic.org/diseases-conditions/hodgkins-lymphoma/symptoms-causes/syc-20352646 Accessed October 10, 2019

[2] Kalhor N Moran C Lymphoproliferative disorders Kalhor N Moran C Mediastinal Pathology Cham Springer International Publishing 2019 521 577 10.1007/978-3-319-98980-8_13

[3] Zapater E Bagán JV Carbonell F Basterra J Malignant lymphoma of the head and neck Oral Dis 2010 16 2 119 128 10.1111/j.1601-0825.2009.01586.x 20374502

[4] Hudnall SD Pathology of Hodgkin Lymphoma Hudnall SD Küppers R Precision Molecular Pathology of Hodgkin Lymphoma. Molecular Pathology Library Cham Springer International Publishing 2018 13 34 10.1007/978-3-319-68094-1_2

[5] Gopas J Stern E Zurgil U Reed-Sternberg cells in Hodgkin’s lymphoma present features of cellular senescence Cell Death Dis 2016 7 11 e2457 e2457 10.1038/cddis.2016.185 27831553 PMC5287295

[6] Adult Hodgkin Lymphoma Treatment (PDQ®)–Patient Version National Cancer Institute https://www.cancer.gov/types/lymphoma/patient/adult-hodgkin-treatment-pdq. Published August 2, 2019 Accessed October 13, 2019

[7] Mandell G Lantieri R Goodman L Tracheobronchial compression in Hodgkin lymphoma in children Am J Roentgenol 1982 139 6 1167 1170 10.2214/ajr.139.6.1167 6983262

[8] Patil V Airway emergencies in cancer Indian J Crit Care Med 2007 11 1 10.4103/0972-5229.32435

[9] Yang F-F Gao R Miao Y Primary tracheobronchial non-Hodgkin lymphoma causing life-threatening airway obstruction: a case report J Thorac Dis 2015 7 12 E667 E671 10.3978/j.issn.2072-1439.2015.12.05 26793387 PMC4703689

[10] Nasal intubation • LITFL Medical Blog • CCC Cardiology Life Fast Lane • LITFL • Med Blog January 2019 https://litfl.com/nasal-intubation/ Accessed March 22, 2020

[11] Yoo H Choi JM Jo J Lee S Jeong S-M Blind nasal intubation as an alternative to difficult intubation approaches J Dent Anesth Pain Med 2015 15 3 181 184 10.17245/jdapm.2015.15.3.181 28879278 PMC5564177

