# Supplementary figures and images for: Rapid Airway Narrowing Associated with Hodgkin’s Lymphoma, a Case Report
Source: J Educ Teach Emerg Med. 2020 Apr 15;5(2):V11–3. doi: 10.21980/J86D3Q (PMC10332571; doi:10.21980/J86D3Q)

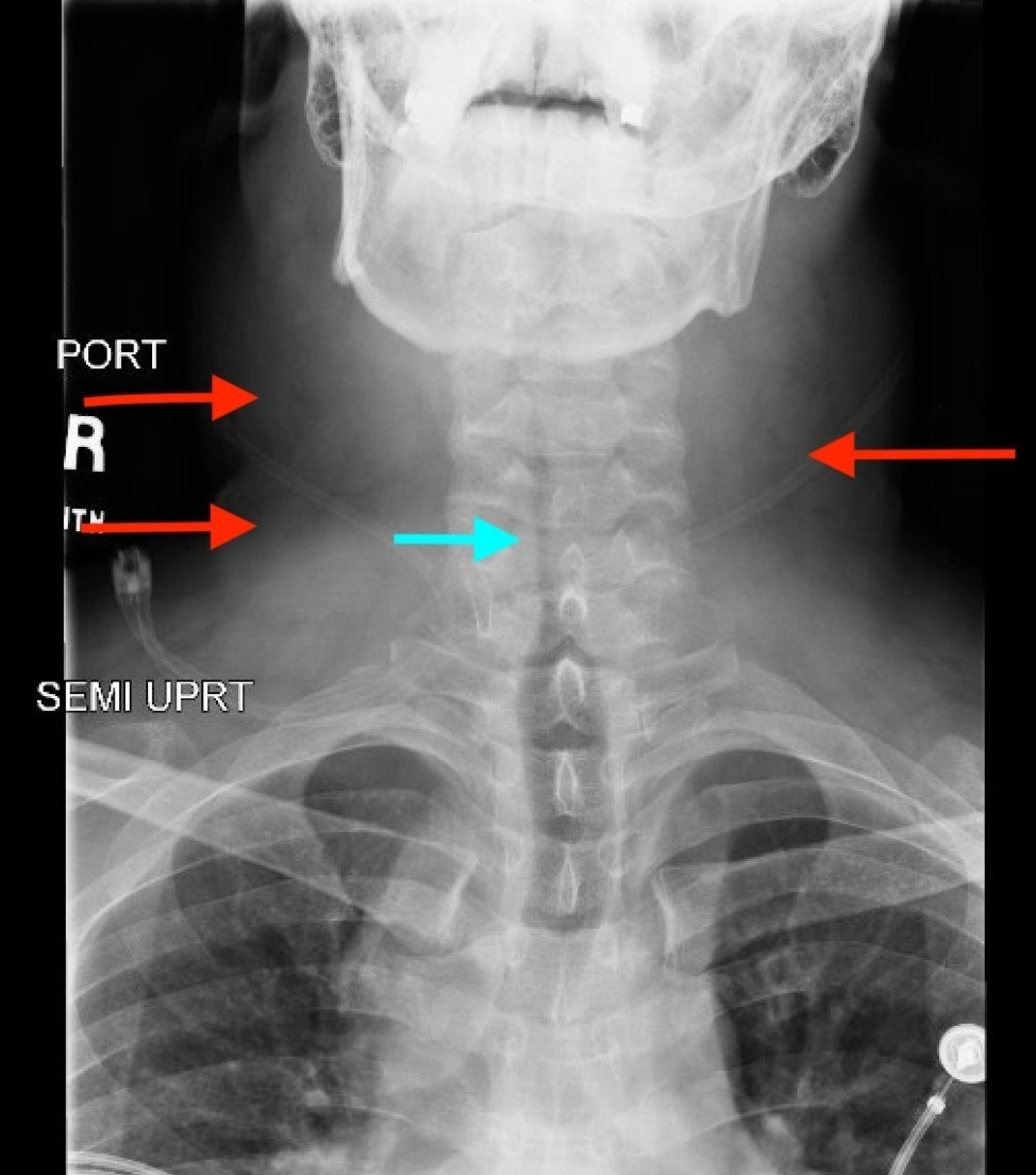

Supplement: Supplementary file 1 [file jetem-5-2-v11-supp1.jpg]

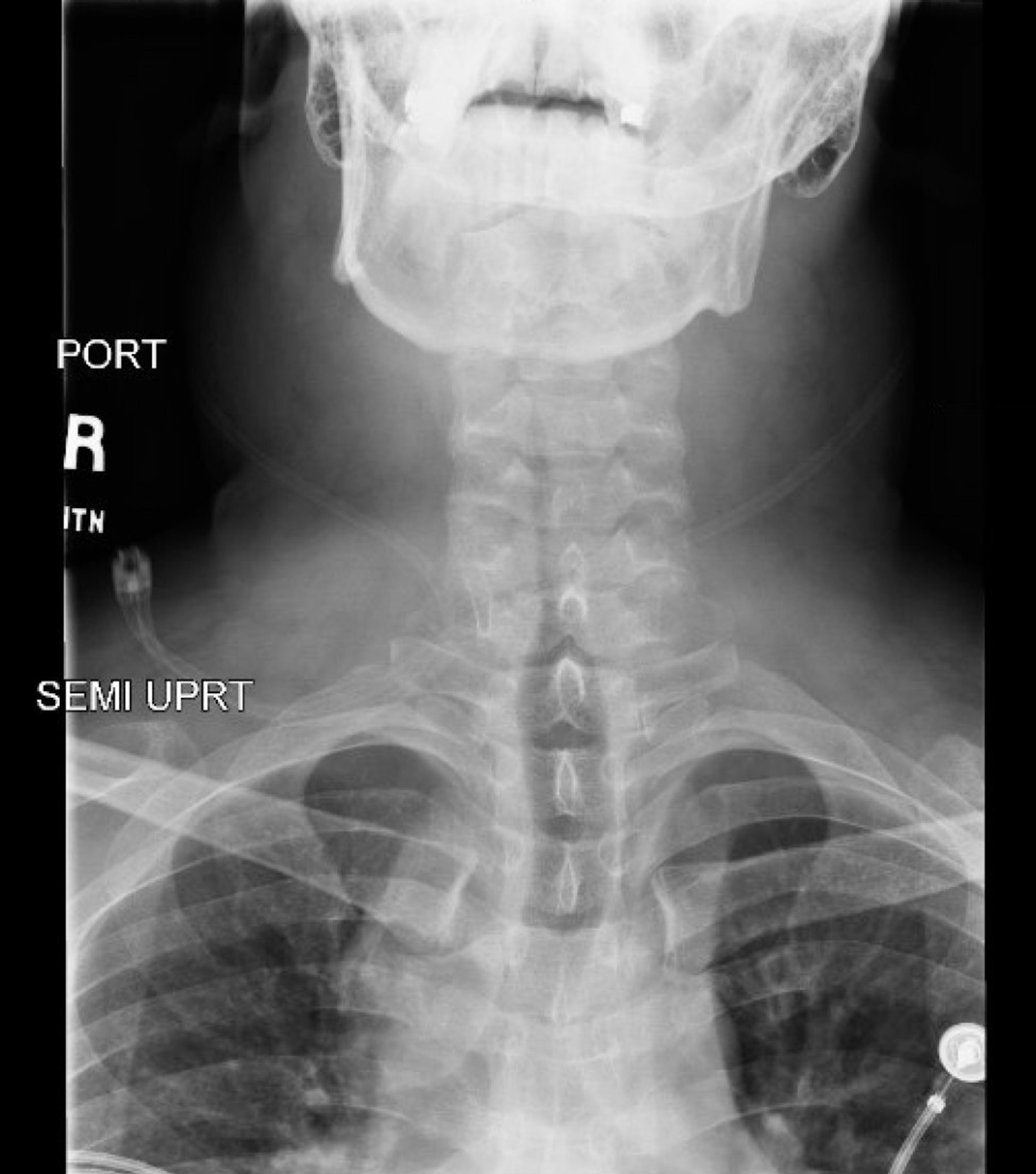

Supplement: Supplementary file 2 [file jetem-5-2-v11-supp2.jpg]

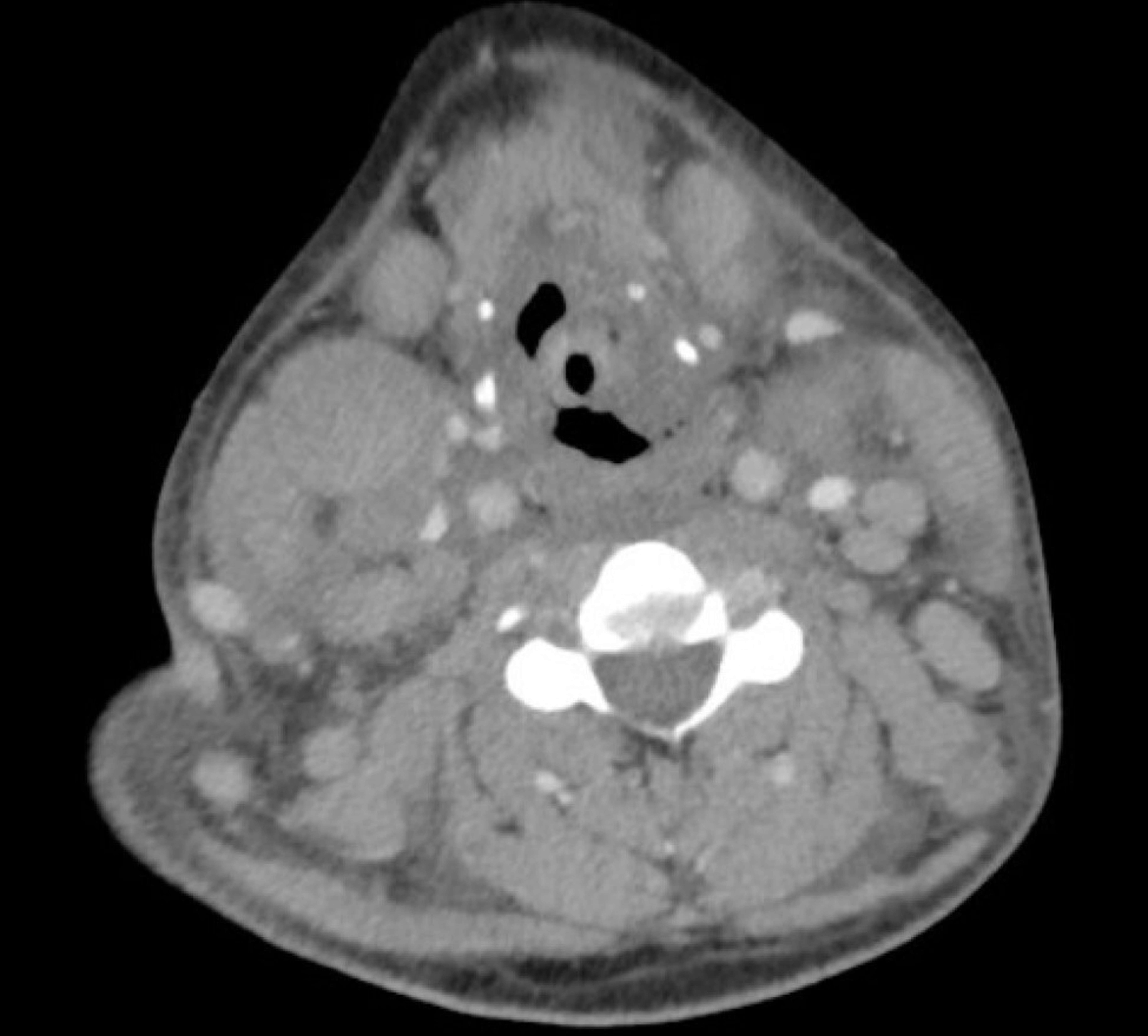

Supplement: Supplementary file 4 [file jetem-5-2-v11-supp4.jpg]

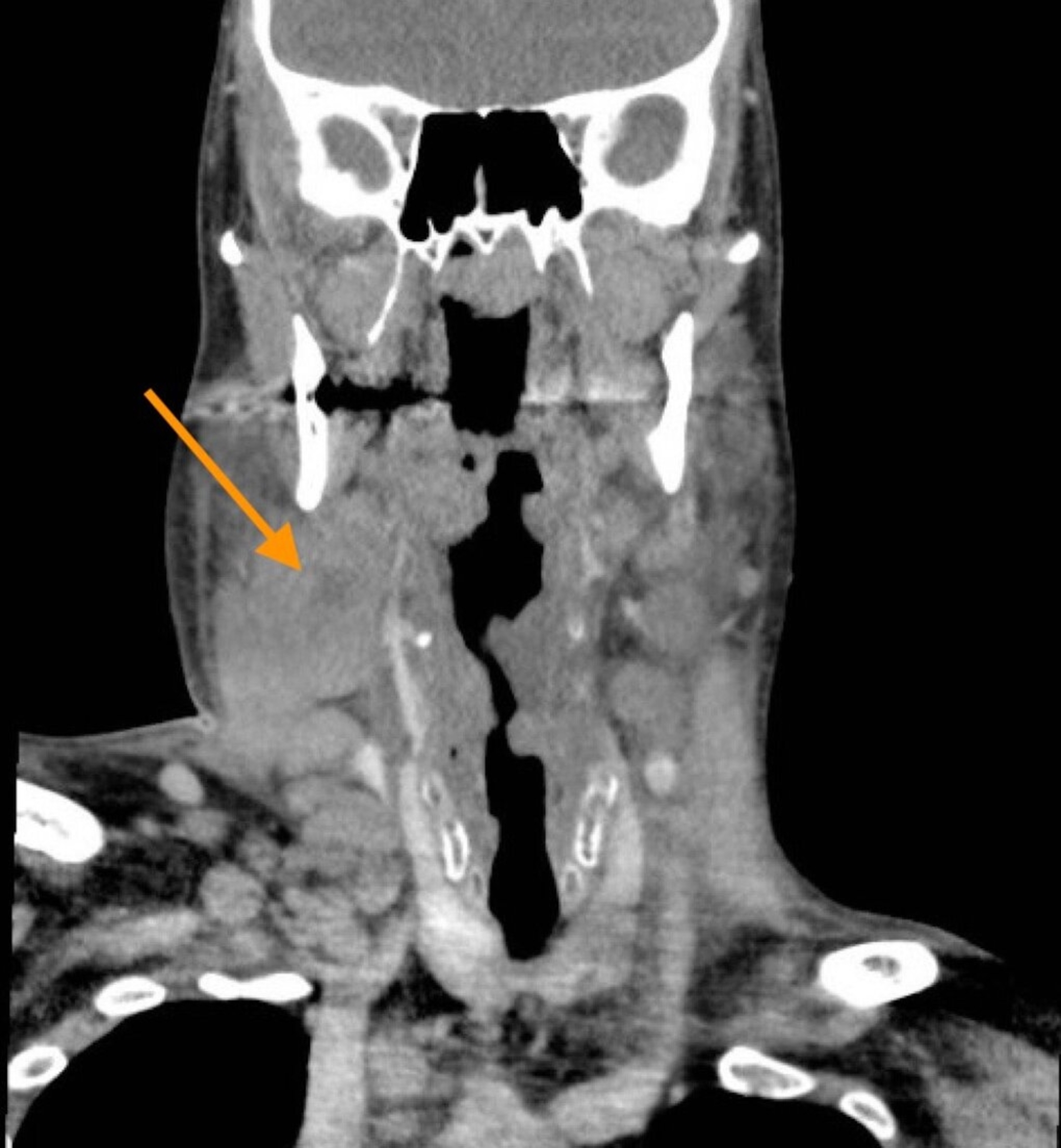

Supplement: Supplementary file 5 [file jetem-5-2-v11-supp5.jpg]

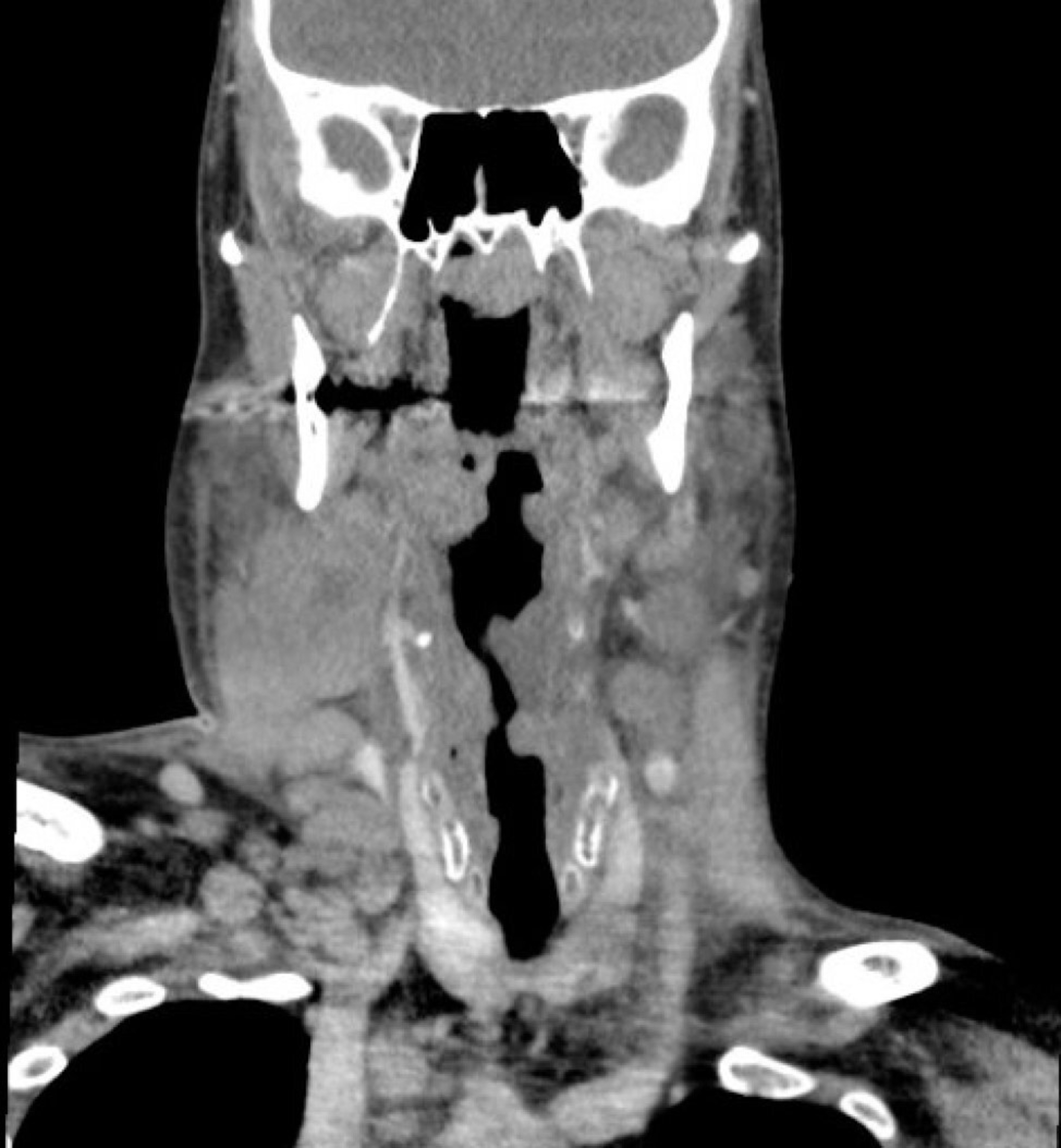

Supplement: Supplementary file 6 [file jetem-5-2-v11-supp6.jpg]

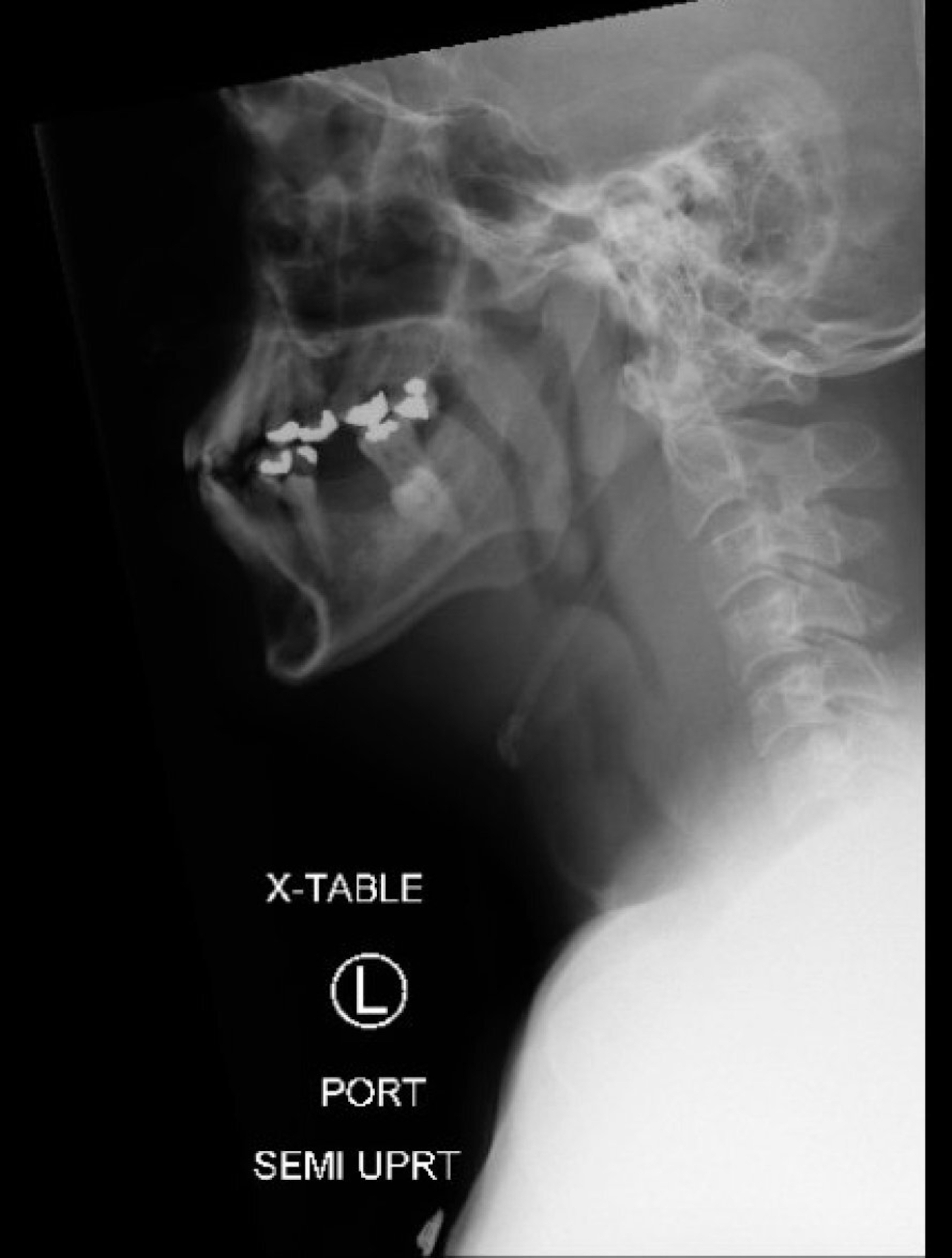

Supplement: Supplementary file 8 [file jetem-5-2-v11-supp8.jpg]
